# Magnitude, outcome, and associated factors of anti-tuberculosis drug-induced hepatitis among tuberculosis patients in a tertiary hospital in North Ethiopia: A cross-sectional study

**DOI:** 10.1371/journal.pone.0241346

**Published:** 2020-11-10

**Authors:** Liwam Kidane Gezahegn, Ermias Argaw, Belete Assefa, Azeb Geberesilassie, Mengistu Hagazi

**Affiliations:** 1 School of Medicine, College of Health Sciences, Mekelle University, Mekelle, Ethiopia; 2 School of Public Health, College of Health Sciences, Mekelle University, Mekelle, Ethiopia; Cleveland State University, UNITED STATES

## Abstract

**Background:**

Short-course chemotherapy comprising isoniazid, rifampicin, ethambutol, and pyrazinamide has proved to be highly effective in the treatment of tuberculosis (TB). However, the most common adverse effect of this regimen leading to interruption of therapy is hepatotoxicity. There is a paucity of evidence in Tigray region on anti-tuberculosis drug-induced hepatitis. Therefore, this study aims to determine the magnitude, outcomes, and associated factors of drug-induced hepatitis in Ayder specialized comprehensive hospital tuberculosis clinic.

**Methods:**

An institution-based cross-sectional study was done on 188 cases of patients who took anti-tuberculosis drugs from August 4, 2015 to June 30, 2018 in tuberculosis clinic, Ayder Comprehensive Specialized Hospital, Northern Ethiopia. Data on socio-demography, clinical characteristics and magnitude of the incidence and outcome of anti-tuberculosis drugs-induced hepatitis were collected using structured checklist from patients’ records using census method. Then, we entered and analyzed the data using statistical packages for social sciences (SPSS) statistical software version 21. Descriptive statistics were done in tables, counts, proportions, median and range. Bivariate and multivariable regression analyses were done to identify factors that are associated with drug-induced hepatitis. Confidence interval was taken at 95% and p-value of less than 0.05 was used to denote significance.

**Results:**

We approached a total of 226 patients’ records, and we collected data from188 records (83.2%response rate). Anti-tuberculosis drug-induced hepatitis was found in 26 (13.8%) of the patients, of which 3 (11.54%) have died. Using multivariable logistic regression analyses, preexisting liver disease (AOR: 42.01, 95% CI: 4.22–417.49), taking other hepatotoxic drugs (AOR: 23.66, 95% CI: 1.77–314.79), and having lower serum albumin (AOR: 10.55, 95% CI: 2.57–43.32) were found to be significantly associated with the development of anti-tuberculosis drug-induced hepatitis.

**Conclusion and recommendation:**

The incidence of anti-tuberculosis drug-induced hepatitis was high. Patients with low baseline serum albumin, taking other hepatotoxic drugs and having preexisting liver disease should be followed with serial liver enzymes after initiation of anti-tuberculosis medications. These patients should be followed with frequent measurement of liver enzymes to assess for the development of drug-induced hepatitis.

## Background

Tuberculosis continues to be a major health problem in both developing and developed countries because of its resurgence among immunosuppressed patients [1–3]. Short-course chemotherapy comprising isoniazid (H), rifampicin (R), ethambutol (E), and pyrazinamide (Z) has proved to be highly effective in the treatment of TB (tuberculosis). However, the most common adverse effect leading to interruption of therapy is hepatotoxicity [4]. Multiple studies done in different countries reported that up to a quarter (2.55%- 36.75%) of patients taking anti-tuberculosis medications develop drug-induced hepatitis during the course of anti TB [5–13]. Anti-TB drug–induced hepatotoxicity is associated with a mortality of 6%– 12% if these drugs are continued after the onset of symptoms [14], and sometimes it can be as high as 22.7% [15]. There are many mechanisms for pathogenesis of drug-induced liver injury, but mostly the exact mechanism remains unclear. These include direct toxicity of the compound or its metabolite, free radicals, immune-mediated injury, and hypersensitivity reactions [16].

The occurrence of drug-induced hepatotoxicity is unpredictable, but it is observed that certain patients are at a relatively higher risk than other populations. Older age, female sex, chronic alcoholism and chronic liver disease, hepatitis B virus carrier status, acetylator status and nutritional status have all been incriminated as possible predisposing factors [17–20]. But data from different countries do not show consistent results, and therefore, it has been difficult to identify patients with increased risk of drug-induced hepatitis.

In Ethiopia, there are few studies of anti-tuberculosis induced hepatitis. Furthermore, there is a paucity of evidence in Tigray region on anti-tuberculosis drug-induced hepatitis. Therefore, this study aims to determine magnitude, outcome, and associated factors of drug-induced hepatitis in Ayder specialized comprehensive hospital tuberculosis clinic.

## Methods and materials

### Study design and setting

The study was conducted from May 1, 2018 to July 15, 2018 at Ayder comprehensive specialized hospital, located in Mekelle, capital of Tigray region, northern part of Ethiopia. It is the only tertiary care hospital in the region which gives service to more than 9 million people in its catchment area including the whole Tigray, some parts of Afar, North-eastern parts of Amhara region and Eritrean refugees. Tuberculosis (TB) diagnosis and treatment is one of the services given in the hospital. The TB clinic which is part of the infectious diseases’ referral clinic has been functioning since 2008. It is run by one nurse and one internist.

### Study design

An institution-based retrospective cross-sectional study design.

### Inclusion and exclusion criteria

All patients’ medical records who took anti TB medications at TB clinic, Ayder comprehensive specialized hospital from August 4, 2015 to June 30, 2018 were included. Patients who are below 18 years old, patients with incomplete clinical data, patients with incomplete investigations and patients who developed elevated liver enzymes and bilirubin while on treatment and in whom the elevation is explained with abnormal hepato-biliary imaging finding were excluded from this study.

### Sample size and sampling procedure

The assumptions used to calculate the actual sample size are: 95% level of confidence with 0.05 α value which yields Z α/2 = 1.96 on the standard normal distribution curve, 5% margin of error and using the incidence of anti-TB drug-induced Hepatitis 11.5% from a study done in Jimma, Ethiopia [21], to estimate the sample size using a single population proportion formula: n = 1.96^2^ x Pex (1- Pex)/d^2^, where n = number of sample size: d = absolute precision (5%): Pex = Expected prevalence of anti-tuberculosis induced hepatitis taken as 11.5%. Using this formula, we found a minimum sample size of 156. However, we censused a total of 226 patients who were treated during the study period, of which, 188 patients fulfilled the inclusion criteria.

### Variables

#### Dependent variables

The dependent variables are the incidence of anti-tuberculosis drug-induced hepatitis and outcome of drug-induced hepatitis.

#### Independent variables

The independent variables are socio-demographic characteristics (Age, Gender), type of tuberculosis (extrapulmonary, pulmonary or both), diagnosis modality, severity of the tuberculosis, underlying chronic liver disease, chronic kidney disease, HBV infection status, HCV infection status, HIV infection status, CD4 count, having stage 4(AIDS) defining illness other than tuberculosis, using other hepatotoxic drugs, serum albumin, and baseline liver enzymes.

### Operational definition

**TB regimen:** In Ethiopia, the first line anti-TB drug formulations for adults are given in a fixed dose regimen, with HRZE (75mg/150mg/400mg/275mg) for intensive phase and HR (75mg/150mg) for continuation phase. The dose for adults is based on patients’ weight band. Patients weighting 20-30kg take 1½tablet, patients weighting 30–39 kg take 2 tablets, patients weighting 40–54 kg take 3 tablets, and patients weighting 55kg and above take 4 tablets of the fixed dose regimen [22].

**Drug induced hepatitis:** Is defined based on American thoracic society’s definition, if ALT and AST are at least 3 times the upper limit of the normal when symptoms of hepatitis are present or at least 5 times the upper limit of the normal if no symptoms of hepatitis are present [17, 23].

**Sever tuberculosis:** central nervous system (CNS) TB, pericardial TB, adrenal TB and miliary TB.

**Mild drug induced hepatitis:** Liver enzyme elevation 3–5 times [24].

**Moderate drug induced hepatitis:** Liver enzyme elevation 5–10 times [24].

**Severe drug induced hepatitis:** Liver enzyme elevation > 10 times [24].

Normal laboratory values based on ACSH laboratory’s reference range:

**Normal AST:** For male up to 37 U/L and female up to 31
**Normal ALT:** For male up to 42 U/L and for female 32
**Normal bilirubin:** Total bilirubin 0.2–1.2 mg/dl and direct fraction of 0.2 mg/dl.

**Lower serum albumin:** Clinically significant low albumin level, less than 3.0 g/dl [25].

### Data collection instrument and technique

Data were collected using structured checklist prepared in English and designed to meet the study objectives. The data were collected from patients’ charts. The checklist contained socio-demographic characteristics, disease-related factors, patient factors, including co-morbidities and outcome questions [6, 12, 26, 27].

Data collection was carried out using two data collectors who were selected from physicians of internal medicine and were given important training which guided them in collecting the appropriate data. Content validity of the checklist was assessed using experts and meanings of all items were checked accordingly.

### Data quality assurance

The checklist was also provided in the original language of English, avoiding the need for translation, and maintaining consistency of the questions and responses. Pre-test was conducted in 10% of the sample size to check the consistency of the checklist.

### Data analysis

The collected data were checked for completeness before the analysis process began. The checked data were coded and entered into Microsoft excel spreadsheet. The coded data were transferred to statistical packages for social sciences (SPSS) version 21. Descriptive statistics were done in tables, counts, proportions, median and range. Association of the independent variables with that of occurrence of anti-tuberculosis drugs induced hepatitis was checked using Bivariate and multivariable logistic regression. The statistical significance was determined at P value less than 0.05 and 95% confidence interval (CI).

### Ethical consideration

Ethical clearance was obtained from Ethical Review Committee of Mekelle University, College of health science institutional review board (IRB). Prior to data collection, official letter was obtained from department of internal medicine in order to get permission from the medical director and research office. The name of the patients was not mentioned and the entire information was kept for patient confidentiality.

## Result

### Socio-demography

We approached a total of 226 patients’ records, and we collected data from 188 records (83.2% response rate). About 106 (56.4%) were males, about 33.5% aged between 18–29 years (Table 1).

**Table 1:** Socio-demographic characteristics of study participants in Ayder comprehensive specialized hospital TB clinic, from August 4, 2015 to June 30, 2018 (N = 188).

Variable	Frequency	Percent (%)
**Sex**		
Male	106	56.4
Female	82	43.6
**Age**		
18–29	63	33.5
30–39	45	23.9
40–49	37	19.7
50–59	21	11.2
≥60	22	11.7

Around 41% of the patients had both pulmonary and extrapulmonary tuberculosis, 62.8% of the patients’ diagnosis of tuberculosis were made using clinical evidence and radiologic modality. Around 80% of the patients had mild to moderate form of tuberculosis; about one-third used other hepatotoxic drugs. One-third of the patients had HIV co-morbidity, of these, 70.9% had stage 4 defining illness other than the tuberculosis; about three-forth had CD4 count less than 200. Around 26.6% had low serum albumin, 6.4% had pre-existing liver disease (cirrhosis, and moderate to severe fatty liver disease). About 81.9% of the patients had normal baseline liver enzymes, and 2.7% had Hepatitis B virus infection (Table 2).

**Table 2:** Disease characteristics and co-morbidity of study participants in Ayder comprehensive specialized hospital TB clinic, from August 4, 2015 to June 30, 2018 (N = 188).

Variable	Frequency	Percent (%)
**Type of TB**		
Extrapulmonary	70	37.2
Pulmonary	41	21.8
Both	77	41.0
**Diagnosis Modality**		
Bacteriological	28	14.9
Histo-pathology	40	21.3
Radiology and Clinical	118	62.8
Clinical	2	1.1
**Severe Tuberculosis**		
No	150	79.8
Yes	38	20.2
Hepatotoxic Drugs*		
No	131	69.7
Yes	57	30.3
**HIV Status**		
Positive	55	29.3
Negative	113	60.1
Unknown Status	20	10.6
**Stage 4 Defining Illness (n = 55)**		
No	16	29.1
Yes	39	70.9
**CD4 Count (n = 55)**		
<200cells/mm^3^	41	74.5
≥200 cells/mm^3^	14	25.5
**Serum Albumin**		
Low	50	26.6
Normal	122	64.9
Not Done	16	8.5
**Abdominal Ultrasound**		
Pre-existing liver disease	12	6.4
No features of pre-existing liver disease	154	81.9
Not done	22	11.7
**Baseline Liver Enzymes**		
Normal	154	81.9
Abnormal	34	18.1
**Hepatitis B Virus Infection**		
Negative	159	84.6
Positive	5	2.7
Not done	24	12.8

*Hepatotoxic drugs: Cotrimoxazole, fluconazole, atrovastatin, valporate, phenytoin, and propylthiouracil.

### Magnitude of drug induced hepatitis

In three years, out of 188 patients, the magnitude of anti-tuberculosis drug-induced hepatitis was 13.8% and median time of onset for development of drug induced hepatitis was 12 days, ranging from 5–52 days. More than two-third of the patients developed moderate to severe type of drug-induced hepatitis and the most common symptom was nausea and vomiting. Eighty eight percent of the patients improved following discontinuation of the anti-tuberculosis drugs while the median time for normalization of the liver enzymes was 14 days, ranging 7–32 days. Among these patients, five had recurrence of drug-induced hepatitis after reinitiating of anti-tuberculosis drugs (Table 3).

**Table 3:** Magnitude of drug-induced hepatitis among study participants in Ayder comprehensive specialized hospital TB clinic, from August 4, 2015 to June 30, 2018 (N = 188).

Variable	Frequency	Percent (%)
**Anti-tuberculosis Drug-induced Hepatitis**		
Yes	26	13.8
No	162	86.2
**Time of Onset of Anti-tuberculosis Drug-induced Hepatitis in Days (n = 26)**		
Median (range)	12 (5–52)	
**Severity of Drug Induced Hepatitis (n = 26)**		
Mild	8	30.8
Moderate	9	34.6
Severe	9	34.6
**Clinical Feature of Patients with Anti-tuberculosis Drug-induced Hepatitis (n = 26)**		
Nausea and vomiting	25	96.2
Malaise	21	80.8
Jaundice	17	65.4
**Outcome of Patients with Anti-tuberculosis Drug-induced Hepatitis (n = 26)**		
Improved after discontinuation	23	88.5
Died	3	11.5
**Time to normalization of liver enzyme after discontinuation of anti-tuberculosis drugs in days (n = 23)**		
Median (range)	14 (7–32)	
**Recurrence of Anti-tuberculosis Drug-induced hepatitis after reinitiating (n = 23)**		
No	18	78.26
Yes	5	21.7

### Bivariate analysis

On bivariate analysis, both descriptive analyses using Chi square and inferential analyses using binary logistic analysis was done. Type of tuberculosis, Severity of tuberculosis diagnosis, taking other hepatotoxic drugs, having preexisting chronic liver disease, HIV infection, lower serum albumin level, and abnormal baseline liver enzymes were significantly associated with the development of drug-induced hepatitis (Table 4).

**Table 4:** Bi-variate analysis of determinants of anti-tuberculosis drug-induced hepatitis in Ayder comprehensive specialized hospital, TB clinic, from August 4, 2015 to June 30, 2018.

Variable	COR	CI		P value
		Lower bound	Upper bound	
**Sex**				
Male	1.12	0.49	2.58	0.78
Female	1			
**Age**				
<60	1			
≥60	1.45	0.45	4.69	0.53
**Type of Tuberculosis**				
Extrapulmonary	1			
Pulmonary	1.83	0.50	6.76	0.143
Both	3.81	1.31	11.06	0.01
**Diagnosis Modality**				
Bacteriological	1			
Radiology and clinical	0.49	0.15	1.56	0.23
Clinical	4.40	0.91	21.24	0.06
Histo-pathology	0.59	0.15	2.28	0.45
**Severe Tuberculosis**				
No	1			
Yes	4.48	1.86	10.78	0.001
**Preexisting Liver Disease**				
No	1			
Yes	9.95	2.86	34.51	0.0001
**Other Hepatotoxic Drugs**				
No	1			
Yes	15.24	5.36	43.31	0.0001
**HIV Status**				
Positive	7.99	3.10	20.57	0.0001
Negative	1			
**Stage 4 Defining Illness**				
No	1			
Yes	1.40	0.40	4.80	0.59
**CD4 Count**				
<200 cells/mm^3^	2.24	0.66	7.54	0.19
≥200 cells/mm^3^	1			
**Low Serum Albumin**				
No	1			
Yes	17.06	5.97	48.72	0.000
**Hepatitis B Virus Infection**				
No	1			
Yes	1.34	0.14	12.49	0.79
**Abnormal Baseline Liver Enzymes**				
No	1			
Yes	4.43	1.81	10.84	0.001

### Multivariable analysis

In multivariable logistic regression analyses, having preexisting chronic liver disease, taking other hepatotoxic drugs and lower serum albumin were found to be independent predictors of developing drug-induced hepatitis. Patients with preexisting liver disease had 42.01 times higher risk of developing DIH (AOR: 42.01, 95% CI: 4.22–417.49). Patients taking other hepatotoxic drugs had 23.66 times higher risk of developing DIH (AOR: 23.66, 95% CI: 1.77–314.79). Patients having lower serum albumin had 10.55 higher risk of developing DIH (AOR: 10.55, 95% CI: 2.57–43.32) (Table 5).

**Table 5:** Multivariable regression of determinants of anti-tuberculosis drug-induced hepatitis in Ayder comprehensive specialized hospital TB clinic, from August4, 2015 to June 30, 2018.

Variable	COR	CI		P value
		Lower bound	Upper bound	
**Type of Tuberculosis**				
Extrapulmonary	1			
Pulmonary	1.11	0.23	5.29	0.89
Both	1.62	0.26	9.84	0.60
**Severe Tuberculosis**				
No	1			
Yes	0.75	0.18	3.08	0.69
**Preexisting Liver Disease**				
No	1			
Yes	42.01	4.22	417.49	0.001
**Other Hepatotoxic Drugs**				
No	1			
Yes	23.66	1.77	314.79	0.01
**HIV Status**				
Positive	0.58	0.05	6.29	0.65
Negative	1			
**Low Serum Albumin**				
No	1			
Yes	10.55	2.57	43.32	0.001
**Abnormal Baseline Liver Enzymes**				
No	1			
Yes	1.53	0.41	5.60	0.52

## Discussion

The magnitude of drug induced hepatitis was found to be 13.8%. Having preexisting chronic liver disease, taking other hepatotoxic drugs and lower serum albumin were found to be independent predictors of developing drug-induced hepatitis.

In this study, the magnitude of drug induced hepatitis was found to be high. Similar results were found in studies done in Pakistan, Egypt and Jimma, Ethiopia which were in the range of 11.5–15% [21, 28, 29]. This could be due to similar socio-demographic and economic background of the patients. In contrast to our finding, studies done in Malaysia, Nepal and Dawro Zone (South Ethiopia) showed the magnitude of DIH to be slightly lower, in the range of 8–9.4% [30–32]. This could be in part because the studies in Dawro (Ethiopia) and in Nepal were prospective cohort studies, and in the Malaysia the sample size was higher. In contrary to the current study, studies done in China and India indicated that the incidence to be very low, 2.55 and 3.8 respectively [5, 6]. The reason for this difference could be because these studies had a large sample size which was 3900 and 4304, respectively, and both were prospective studies.

The median time of development of the drug-induced hepatitis was 12 days, ranging 5–52 days. Other studies done in Pakistan, Egypt and Dawro zone (Ethiopia) had similar results [29, 31, 32].

The most common clinical features were nausea and vomiting (96.2%) followed by malaise and jaundice, 80.8% and 65.4%, respectively. This finding was in line with other studies in Jimma, (Ethiopia), Dawro Zone (Ethiopia), and Egypt [9, 21, 32]. This could be due to similarity of clinical features between acute viral hepatitis and drug-induced hepatitis.

In our study, we found that 34.6% of the patients had severe drug-induced hepatitis, which is similar with a study done in Jimma, Ethiopia [21].

In our study, age and sex were not significantly associated with development of DIH. Similar studies done in Iran and India also showed no statistically significant association between DIH and sex or age of the patients [26, 33]. In contrast, another study done in India found that patients with age ≥60 years and female gender were significantly associated with drug-induced hepatitis [6]. This could be due to participants in the Indian study were older as compared to our study, in which most of our study participants were younger age groups.

In our study, type of tuberculosis, severity of the tuberculosis, and diagnostic modality were not significantly associated with drug-induced hepatitis. In contrast, in two studies done in India and one study in Jimma, Ethiopia showed that having extensive disease was significantly associated with the development of drug induced hepatitis [6, 21, 34]. In contrast to our study, in a study in India unconfirmed tuberculosis was significantly associated with development of DIH [35]. The reason for this could be the low number of patients who have definite diagnosis of tuberculosis in our study participants.

In our study, preexisting liver disease and use of other hepatotoxic drugs were significantly associated with DIH. Inline to the current study, use of other hepatotoxic drugs and preexisting liver disease were significantly associated with study done in Iran and India respectively [33, 35].

In this study, abnormal baseline liver enzyme was not significantly associated with DIH. Unlike our study, abnormal baseline liver enzyme was significantly associated with DIH in study done Iran [33].

This study showed that being HIV positive, lower CD4 count and stage 4 defining illness were not significantly associated with development of anti-tuberculosis drug-induced hepatitis. In contrary to our study, studies in Iran and Malaysia found HIV to be risk factor for DIH [33, 36]. The low number of HIV infected patients in the Malaysian study as compared to our study may explain this difference; in addition, the difference may be due to difference in study design as the Malaysian study was case-control.

In our study, having a positive HBsAg result was not significantly associated with development of drug-induced hepatitis. Similar to our study, in a study done in Malaysia hepatitis virus infection was not associated with development of DIH. This may be due to the small number of patients with Hepatitis virus infection in both studies [30]. In contrast to our study, in a study done in India, hepatitis B infection was a significant risk factor for development of DIH [35]. This difference could be explained by the very low number of patients with HBsAg positive results in our study participants.

In our study participants, lower serum albumin was significantly associated with development of drug-induced hepatitis. This is in-line with studies done in India, Egypt and Malaysia [6, 29, 34, 36].

In our study, we found that among the patients who developed anti-tuberculosis drug-induced hepatitis, 23(88.5%) patients improved with discontinuation of the anti-tuberculosis treatment, and 3 (11.5%) patients died. Similarly, in a study done in China it was found that 3.77% died as result of DIH and 96.23% improved with drug discontinuation [5]. Similar results were also found in a study done in Egypt [29].

In our study, no patient developed acute liver failure. In contrast to our study, a study done in India found that one-fourth of the patients developed serious complications, such as fulminant and subacute hepatic failure [35]. The difference might be because our study population was on direct observed therapy; therefore, early detecting of the DIH and discontinuation of the drugs might contribute to this low incidence of acute liver failure.

The median time to normalization of liver enzymes was 14 days, ranging from7-30 days. Similar results were found in a study done in Egypt [29]. In our study, recurrence of drug-induced was observed in 5 (21.7%) of the patients, which were similar with studies done in India and New York [6, 27].

## Limitations of the study

Important variables, like alcohol intake and body mass index were not included in the study due to the retrospective nature of the study. Another limitation was the small sample used; therefore, generalizing the result is difficult.

## Conclusion

In this study the magnitude of drug induced hepatitis was found to be high. Lower serum albumin, taking other hepatotoxic drugs, and having preexisting chronic liver disease were found to be independent predictors of drug-induced hepatitis. There was high rate of recurrence and mortality.

## Supporting information

S1 Dataset (SAV)

S1 File (SAV)

## References

[1] Kasper D, fauci A, Hauser S, et al Harrison’s principles of internal medicine. New York: McGraw-hill medical, 2015, 19th.

[2] Mandel GL, Bennett JE, Dolin R. Mandell, Douglas, and Bennett’s principles and practice of infectious diseases. United States: Natasha Andjelkovic, 2010, 7th.

[3] Kebede A, Alebachew Z, Tsegaye F, et al First Ethiopian national population based tuberculosis prevalence survey population based. Federal democratic republic of Ethiopia ministry of health 2011.

[4] Schaberg T, Rebhan K, Lode H. Risk factors for side effects of isoniazid rifampicin and pyrazinamide in patients hospitalized for pulmonary tuberculosis. Eur Respir J 1996; 9:2026–2030. 10.1183/09031936.96.09102026 8902462

[5] Shang P, Xia Y, Liu F, Wang X, Yuan Y, et al Incidence, Clinical Features and Impact on Anti-Tuberculosis Treatment of Anti-Tuberculosis Drug Induced Liver Injury (ATLI) in China. PLoS ONE 2011; 6(7): e21836 10.1371/journal.pone.0021836 21750735 PMC3130045

[6] Gaude GS, Chaudhury A, Hattiholi J. Drug induced hepatitis and the risk factors for liver injury in pulmonary tuberculosis patients. J Family Med Prim Care 2015; 4(2): 238–243 10.4103/2249-4863.154661 25949974 PMC4408708

[7] Mekonnen T, Abseno M, Meressa D, Fekde B. Prevalence and management out comes of anti TB drugs induced hepatotoxicity, St.Peter TB Specialized Hospital. J Ethiopia Med Pract. 2002;4(1): 32–38

[8] Parthasarathy R, Sarma GR, Janardhanam B, et al Hepatic toxicity in South Indian patients during treatment of tuberculosis with short-course regimens containing isoniazid, rifampicin and pyrazinamide. Tubercle 1986; 67:99–108. 10.1016/0041-3879(86)90003-6 3775870

[9] Türktaş H, Ünsal M, Tülek N, Örüç O. Hepatotoxicity of antituberculosis therapy (rifampicin, isoniazid and pyrazinamide) or viral hepatitis. Tubercle Lung Dis 1994; 75:58–60. 10.1016/0962-8479(94)90104-X 8161767

[10] Combs DL, O’Brien RJ, Geiter LJ. USPHS tuberculosis short course chemotherapy trial 21: Effectiveness, toxicity and acceptability. The report of final results. Ann Intern Med 1990; 112:397–406. 10.7326/0003-4819-76-3-112-6-397 2155569

[11] Quyen T. Management of common side effects of INH (Isoniazid), RIF (Rifampin), PZA (Pyrazinamide), and EMB (Ethambutol).2008

[12] Lima MF, Melo HR. Hepatotoxicity induced by anti-tuberculosis drugs among patients co infected with HIV and tuberculosis. Cad Saude Publica 2012; 28: 698–708. 10.1590/s0102-311x2012000400009 22488315

[13] British Thoracic Society and Tuberculosis Association. Short course chemotherapy in pulmonary tuberculosis. Lancet 1975; 305:119–24 46047

[14] Dash LA, Comstock GW, Flynn PG. Isoniazid preventive therapy: retrospect and prospect. Am Rev Respir Dis 1980; 121:1039–1044. 10.1164/arrd.1980.121.6.1039 7416591

[15] Devarbhavi H, Singh R, Patil M, Sheth K, Adarsh CK, Balaraju G. Outcome and determinants of mortality in 269 patients with combination anti-tuberculosis drug-induced liver injury. JGastroenterol Hepatol 2013; 28: 161–7. 10.1111/j.1440-1746.2012.07279.x 23020522

[16] Brewer TF, Heymann SJ. To control and beyond: moving towards eliminating the global tuberculosis threat. J Epidemiol Community Health, 2004; 58:822–825. 10.1136/jech.2003.008664 15365106 PMC1763333

[17] Saukkonen JJ, Cohn DL, Jasmer RM, Schenker S, Jereb JA, Nolan CM, et al An offiial ATS statement: Hepatotoxicity of antituberculosis therapy. Am J Respir Crit Care Med, 2006; 174:935–52. 10.1164/rccm.200510-1666ST 17021358

[18] Tostmann A, Boeree MJ, Aarnoutse RE, de Lange WC, van der Ven AJ, et al Anti-tuberculosis drug induced hepatotoxicity: Concise uptodate review. J Gastroen Heaptol 2008; 23: 192–202. 10.1111/j.1440-1746.2007.05207.x 17995946

[19] Singh J, Arora A, Garg PK. Anti-TB treatment induced hepatotoxicity: role of predictive factors. Postgraduate Medicine Journal 1995; 71: 359–362. 10.1136/pgmj.71.836.359 PMC2398146 7644398

[20] Pande JN, Singh SP, Khilnani GC, Khilnani S, Tandon RK. Risk factors for hepatotoxicity from anti-tuberculosis drugs: A case-control study. Thorax 1996; 51:132–6. 10.1136/thx.51.2.132 8711642 PMC473016

[21] Hassen A, Belachew T, Yami A, Ayen WY. Anti-Tuberculosis Drug Induced Hepatotoxicity among TB/HIV Co-Infected Patients at Jimma University Hospital, Ethiopia: Nested Case-Control Study. PLoS ONE 2013;8:e64622 10.1371/journal.pone.0064622 23696901 PMC3655990

[22] Federal democratic republic of Ethiopia ministry of health: National comprehensive tuberculosis, leprosy and TB/HIV training manual for health care workers. 2016.

[23] Benichou C. Criteria of drug-induced liver disorders. Report of an international consensus meeting. J Hepatol 1990; 11: 272–6.5. Kaplowitz N. Drug-induced liver injury. Clin Infect Dis 2004; 38 Suppl 2: S44–8. 10.1016/0168-8278(90)90124-a 2254635

[24] Variane F, Rich M. Tuberculosis practical guide for clinicians nurses laboratory technicians and medical auxiliaries. Geneva Switzerland: Medecins sans frontiers, 2014^th^ edition.

[25] Freidman LS. uptodate 21.6: Tests of the liver's biosynthetic capacity (eg, albumin, coagulation factors, prothrombin time). 2012.

[26] Ghasemi BR, Haj AM, Samimi R. Drug-induced Hepatitis (Abundance and Outcome during Course of Tuberculosis Treatment): Seven-year Study on 324 Patients with Positive Sputum in Iran. Govaresh 2011; 16(2):134–138

[27] Ungo JR, Jones D, Ashkin D, Hollender ES, Bernstein D, et al Anti-tuberculosis drug-induced hepatotoxicity. The role of hepatitis C virus and the human immunodeficiency virus. Am J Respir Crit Care Med 1998; 157: 1871–1876. 10.1164/ajrccm.157.6.9711039 9620920

[28] Abbasi MA, Ahmed N, Suleman A, et al Common risk factors for the development of anti tuberculosis treatment induced hepatotoxicity. J Ayub Med Coll Abbottabad 2014;26(3). 25671954

[29] Makhlouf HA, Helmy A, Fawzy E, El-Attar M, Rashed HA. A prospective study of antituberculous drug-induced hepatotoxicity in an area endemic for liver diseases. Hepatol Int 2008; 2: 353–360. 10.1007/s12072-008-9085-y 19669265 PMC2716885

[30] Marzuki OA, Fauzi AR, Ayoub S, Kamarul IM. Prevalence and risk factors of anti-tuberculosis drug-induced hepatitis in Malaysia. Singapore Med J 2008; 49: 688–693. 18830542

[31] Shakya R, Shrestha B. Evaluation of risk factors for anti-tuberculosis drugs-induced hepatotoxicity in Nepalese population. Kathmandu Univ J Sci Engg Technol 2006;2(1):1–8

[32] Abera W, Cheneke W, Abebe G. Incidence of anti-tuberculosis drug induced hepatotoxicity and associated risk factors among tuberculosis patients in Dawro Zone, South Ethiopia: A cohort study. International journal of mycobacteriology 2016; 5:14–20 10.1016/j.ijmyco.2015.10.002 26927985

[33] Khalili H, Dashti-Khavidaki S, Rasoolinejad M, Rezaie L, Etminani M. Anti-tuberculosis drugs related hepatotoxicity; incidence, risk factors, pattern of changes in liver enzymes and outcome. DARU 2009; 17: 163–167.

[34] Sharma SK, Balamurugan A, Saha PK, Pandey RM, Mehra NK. Evaluation of clinical and immunogenetic risk factors for the development of hepatotoxicity during anti-tuberculosis treatment. Am J Respir Crit Care Med 2002; 166: 916–919. 10.1164/rccm.2108091 12359646

[35] Anand A.C., K Seth A., Paul M., et al Risk factors for hepatotoxicity during anti-tuberculosis treatment, Med. J. Armed Forces India 2006; 62: 45–49 10.1016/S0377-1237(06)80155-3 27407844 PMC4923276

[36] Fauzi ARM, Shah A, Rathor MY, Satwi S. Risk Factors for Anti Tuberculous Drugs Induced Hepatitis: A prospective survey from a chest clinic in a general hospital. Med J Malaysia 2004; 59:72–7. 15535339

