# Supplementary material for: Magnitude, outcome, and associated factors of anti-tuberculosis drug-induced hepatitis among tuberculosis patients in a tertiary hospital in North Ethiopia: A cross-sectional study
Source: PLoS One. 2020 Nov 10;15(11):e0241346. doi: 10.1371/journal.pone.0241346 (PMC7654771; doi:10.1371/journal.pone.0241346)
Supplement: Attachment — Submitted filename: Response to reviewers.docx [file pone.0241346.s003.docx]

**Rebuttal letter**

**To the academic editor**

1. Please ensure that your manuscript meets PLOS ONE's style requirements, including those for file naming.

**Response**: We followed PLOS ONE's style requirements,

2. In ethics statement in the manuscript and in the online submission form, please provide additional information about the patient records used in your retrospective study. Specifically, please ensure that you have discussed whether all data were fully anonymized before you accessed them and/or whether the IRB or ethics committee waived the requirement for informed consent. If patients provided informed written consent to have data from their medical records used in research, please include this information

**Response:** We have already described that the identification of patients was not mentioned and the entire information was kept for patient confidentiality.

3. PLOS requires an ORCID iD for the corresponding author in Editorial Manager on papers submitted after December 6th, 2016. Please ensure that you have an ORCID iD and that it is validated in Editorial Manager.

**Response:** 0000-0002-1378-0670

4. We note you have included a table to which you do not refer in the text of your manuscript. Please ensure that you refer to Table 3 in your text;

**Response:** We have included “Table 3” in the text of our manuscript

5. Please include captions for your Supporting Information files at the end of your manuscript, and update any in-text citations to match accordingly.

**Response:** We included “S1 dataset” as supporting information.

**To reviewer 1**

1. Small sample size as a cross-sectional study limited the power of the study.

**Response:** comment accepted.

2. More clinical details need to provided. For example,
   a. Details of drug combination and dosing regimen need to be added. This is important because drug-drug interaction is a known factor for DILI of the chemotherapy for TB. The details of drug combination and dosing regimen are critical to understand the risk of DILI due to DDI.

**Response:** We included the following information:

“TB regimen; In Ethiopia, the first line anti-TB drug formulations for adults are given in a fixed dose regimen, with HRZE (75mg/150mg/400mg/275mg) for intensive phase and HR (75mg/150mg) for continuation phase. The dose for adults is based on patients’ weight band. Patients weighting 20-30kg take 1½ tablet, patients weighting 30-39 kg take 2 tablets, patients weighting 40-54 kg take 3 tablets and patients weighting 55kg and above take 4 tablets of the fixed dose regimen”

b. What are "other hepatotoxic drugs" discussed in the manuscript?

**Response:** we included the following information under table 2 as a footnote:

**“*****H**epato-toxic drugs: Cotrimoxazole, fluconazole, atrovastatin, valporate, phenytoin, and p**ropylthiouracil”**

c. What are the "preexisting liver disease" discussed in the manuscript? The severity of the preexisting conditions?

**Response:** we inserted the following information in the text description section of table 2:

“(Cirrhosis, and moderate to severe fatty liver disease)”

3. Language quality of the manuscript need to be improved.
   a. Many spaces are missing cause readability issues.

**Response:** We went through end to end of the manuscript and corrected the formatting and grammatical errors as it is evidenced in the document track changes.

b. Scientific and accurate description is preferred.

**Response:** we tried to follow scientific writing style and we corrected the errors in the table.

**To reviewer 2**

1. Data from Table-2, for calculating frequency and percentage for Stage 4 defining illness and CD4 count, the total participants number N, will be 55 (HIV positive status) and not 188. The calculations were made using N=55; So, the authors are encouraged to mention the N value specifically.

**Response:** corrected accordingly

2. Grammar and spacing errors are present in the manuscript; the authors should carefully proofread the paper for language errors.

**Response:** We went through end to end of the manuscript and corrected the formatting and grammatical errors as it is evidenced in the document track changes.
